# Pouring out the dirty bathwater without throwing away either the baby or its parents: commentary to Saunders et al.

**DOI:** 10.1007/s00247-017-4003-x

**Published:** 2017-10-23

**Authors:** Niels Lynøe, Måns Rosén, Göran Elinder, Boubou Hallberg, Pia Sundgren, Anders Eriksson

**Affiliations:** 1 Stockholm Centre for Healthcare Ethics, Karolinska Institutet, 171 77 Stockholm, Sweden; 2 Department of Learning, Informatics, Management and Ethics, Karolinska Institutet, Stockholm, Sweden; 3 Department of Clinical Science and Education, Södersjukhuset, Karolinska Institutet, Stockholm, Sweden; 4 Department of Clinical Science, Intervention and Technology, Karolinska Institutet and Karolinska University Hospital, Stockholm, Sweden; 5 Department of Diagnostic Radiology, Clinical Sciences, Lund University, Lund, Sweden; 6 Department of Community Medicine and Rehabilitation, Forensic Medicine, Umeå University, Umeå, Sweden

Dear Editors,

We do respect the authors’ deep ambitions to protect vulnerable children exposed to the risk of being abused [1]. We do not, however, understand in what way the Swedish Agency for Health Technology Assessment and Assessment of Social Services (SBU) report “may have disrupted efforts to protect vulnerable children.” The SBU report is an impartial, systematic review of the scientific basis for conclusions regarding the diagnostic accuracy of the triad in relation to traumatic shaking [2, 3]. The main conclusion of the review might have come as a surprise to those who believe the validity of the diagnostic accuracy was high and robust. But how could correct and scientifically based conclusions bring about “catastrophic consequences”? On the contrary, the problem is that the main part of previously published scientific studies is of very low quality regarding evidence [2–4]. The SBU report is merely the messenger of bad news. But trying to kill the messenger by questioning the panel’s scientific competence and the assessment procedures does not change the general picture of badly conducted science [5].

Saunders et al. [1] presented some specific viewpoints that deserve to be commented on.

1. First of all we would like to specify that the SBU report focused on *isolated traumatic shaking,* meaning that there were no other physical signs than subdural hematoma, retinal hemorrhages and encephalopathy — referred to as *the triad*. Hence, we did not include studies whose focus was on infants also suffering from fractures, bruises or other signs of trauma. Even though Saunders et al. [1] admitted that the triad is insufficient for establishing abuse, the isolated triad has been and is still used to classify traumatic shaking cases in scientific studies, as well as to certify abuse in court proceedings. We have previously responded to this issue [6, 7].

2. “As physicians, we do not diagnose *shaking*, we diagnose *abuse*.” We take the opposite standpoint; a medical expert can have a hypothesis of the mechanism behind a medical finding, but to decide whether a trauma was inflicted *intentionally* or *unintentionally* is not a medical issue; this is the task of the judicial system. Exactly how Saunders et al. [1] concluded that a medical finding entails trauma that has been inflicted *intentionally* is not clear; they only stated that the diagnosis is based on “all available data,” often “assessed by a dedicated multidisciplinary team.” This was, however, what was criticized in the SBU report, because it means that the diagnosis is based on preconceptions resulting in circular reasoning [8]. We agree with Saunders et al. that a physician who blinds her/himself to relevant medical information is “unable to meet the standard of care.” But this is not what is criticized in the SBU report; what is criticized is that *scientists* have used unblinded classifications, inducing bias of various forms in previously published research. To imply in a scientific context that such highly relevant critique of previous research was “directly borrowed from lawyers” is unwarranted.

3. Saunders et al. [1] wondered why we did not include the study by Biron and Shelton [9] in the quality assessment. This study indicated that certain cases could have been caused by isolated traumatic shaking because some people had confessed to this. The Biron study, together with others [10, 11], was actually assessed but could only support the second conclusion: that there is *limited* scientific (*low-quality*) evidence that the triad can be associated with traumatic shaking [2, 3] an issue that we have previously responded to [12].

Furthermore, Saunders et al. [1] advanced the criticism that we “ignored papers describing forensic pathology findings in fatally abused children.” We did not. All publications were handled in the same way as declared in the methodological section of the report [2, 3].

4. The aim of the SBU project was to assess the scientific quality of the literature regarding shaken baby syndrome/abusive head trauma in order to examine the diagnostic accuracy of the triad. Certainly, the aim was not to provide “lawyers with new ammunition to question valid data.” The point of departure of the project was the fact that if the diagnostic procedure was not accurate it might have *two* “catastrophic consequences”: (1) an infant who actually should be removed from an unsafe family might risk not being removed and a perpetrator go free; or (2) an infant could be unnecessarily removed, a family split, and an innocent parent convicted.

5. Although we suggested that only confessed or witnessed cases should be applied as *gold standard* when classifying cases and controls, we have also stressed that false confessions might be an issue. In order to minimize the risk of false confessions, we suggested that future studies must examine and specify the circumstances under which a confession was provided, and try to establish whether or not the suspect was confabulating when providing details about what happened.

Furthermore, future studies need to specify the role of any expert witness in this context — did he/she maintain that *all* other “acceptable” explanations had been ruled out, and it was accordingly concluded by default that the infant must have been violently shaken?

6. The latter reasoning is one of the big issues when a member of a child protection team reports to the police and appears as an expert in court. This is an issue because the criteria for classifying shaken baby syndrome cases and controls have become more or less biased. If the parent cannot provide an explanation for why the infant suddenly became unconscious and the isolated triad is detected, this lack of explanation is considered “not acceptable” and the case is classified as a true-positive case. If the parent admits that the baby was shaken *after* it became unconscious, the explanation is also considered “not acceptable” and the case is classified as a true-positive case. If the parent says the infant fell from a certain height (<1 m or <3 m), the explanation is regarded as “not acceptable” and the case is classified as a *true*-*positive case*. In all instances, the parent is assumed by default to be lying [13].

7. The problem is that all these “not acceptable” explanations are associated with certain basic assumptions about the mechanism behind the shaken baby syndrome. For example, the assumption that the presence of the isolated triad presupposes a fall from a certain height is based on theories about the mechanism behind the triad. Also, the default assumption that if the parent cannot provide an “acceptable” explanation then the child must have been violently shaken is based on (the opposition to) certain theories about underlying mechanism. But as demonstrated in the SBU report, the theories about mechanism (derived from biomechanical studies) are contradictory [2]. Hence, relying on one theory about the etiology and pathogenesis while ignoring others is problematic; in order to illustrate why, let us look at an example. We are in a situation similar to the discussion of peptic ulcer before the *Helicobacter pylori* theory eventually was accepted in the 1990s. Earlier on there had been several contradictory hypotheses about the etiology and pathogenesis of peptic ulcer. Typical examples were that it was a psychosomatic disease, or a dysfunction of the gastric acid-producing parietal cells. Common for all theories, however, was the basic assumption that peptic ulcer could not be caused by a microorganism and treatment with antibiotics was deemed “quackery” [14]. Similarly, the reason why we found no studies with the isolated triad disassociated with traumatic shaking was the basic assumption: it is currently considered impossible! Hence, the PIRO (Population, Index test, Reference test/gold standard and Outcome measure) suggested by Saunders et al. [1] could not be applied.

8. We believe that the lack of a generally accepted theory on the etiology and pathogenesis of the triad is *the* major problem behind the different views, interpretations and classifications of traumatic shaking cases and controls. As long as there is no generally accepted theory we should stick to “black box reasoning,” meaning that the focus must be on unbiased observations of different phenomena, looking at input and output without referring to a certain, preconceived mechanism [15]. Accordingly, we must avoid taking for granted that some explanations are “acceptable” and others are not [8]. This means that we should not ignore, e.g., witnessed observations indicating that the triad might have been caused by a minor fall [16]. The reason why some issues have never been investigated is that such observations are against the basic assumptions embraced by child protection teams [13].

Unfortunately, the more premature a mechanism theory is, the higher the risk that the classifications of cases and controls become biased — resulting in an overestimation of *true-positive* cases and an underestimation of *false-positive* cases [8]. A strong belief by an expert witness may also have the “catastrophic consequence” that the court, less knowledgeable of the scientific controversies, is convinced beyond reasonable doubt even when the scientific evidence is actually of low quality.

9. Finally, Saunders et al. [1] correctly stated that several critical comments have followed the publication of the SBU report in *Acta Paediatrica*. Unfortunately, they failed to mention that we have responded to all these comments [6–8, 17–20], and that some of our responses concerned the issues they themselves brought up.
